# Challenging Management of Acute Cholangitis in a Patient With Unextractable Common Bile Duct Stone Post Gastrojejunostomy Surgery

**DOI:** 10.7759/cureus.53561

**Published:** 2024-02-04

**Authors:** Ryan M Lynn, Fady Israel, Steve O Obanor

**Affiliations:** 1 Department of Academic Medicine, New York Institute of Technology College of Osteopathic Medicine, Old Westbury, USA; 2 Department of Gastroenterology, Maimonides Medical Center, Brooklyn, USA

**Keywords:** electrohydraulic lithotripsy, gastrojejunostomy, acute cholangitis, general surgery, acute cholelithiasis

## Abstract

This case report details the management of an 89-year-old female with a complex surgical history, including cholecystectomy, left hepatectomy, and repair of an iatrogenic duodenal perforation, who presented with acute cholangitis due to a large common bile duct stone. Faced with the challenges of altered anatomy and the patient's frailty, a multidisciplinary team employed a series of interventions, including percutaneous transhepatic biliary drainage and endoscopic retrograde cholangiopancreatography (ERCP). The case was further complicated by difficulties in navigating the altered biliary tract, necessitating the use of advanced endoscopic techniques such as cholangioscopy and electrohydraulic lithotripsy (EHL) for stone fragmentation and removal, leading to the successful resolution of the obstruction. This report underscores the importance of individualized care and highlights the efficacy of innovative endoscopic approaches in managing complex biliary disorders in elderly patients with significant surgical histories.

## Introduction

Gallstone disease is one of the most common public health problems in the United States. Approximately 10-20% of the national adult population currently carry gallstones, and gallstone prevalence is rising [1]. Acute biliary infection is a systemic infectious disease which requires prompt treatment and has a significant mortality rate if not addressed in an appropriate manner [2]. This case report explores the intricate management challenges associated with recurrent biliary obstruction in an elderly patient with a complex surgical history [3]. The advanced age and complicated surgical interventions of the patient necessitate a nuanced and multidisciplinary approach to effectively address the recurrent biliary obstruction within the context of altered anatomy and prior biliary-enteric anastomosis [4].

This report sheds light on the complexities encountered in treating such cases, emphasizing the importance of individualized patient care. The discussion delves into the significance of a multidisciplinary strategy and advanced endoscopic techniques, showcasing the delicate balance required in managing these intricate scenarios. The successful resolution of the acute presentation serves as a testament to the effectiveness of a well-coordinated approach, highlighting the need for careful consideration and personalized decision-making when confronting complex medical conditions in elderly patients with a history of multiple surgeries.

## Case presentation

An 89-year-old female patient with a distant history of cholecystectomy and left hepatectomy was previously hospitalized due to retained common bile duct (CBD) stones. Her prior admission was marked by complications, notably iatrogenic duodenal perforation, during an attempted endoscopic retrograde cholangiopancreatography (ERCP). This necessitated emergency exploratory surgery, during which a 1-2 cm anterior duodenal perforation was repaired using a Graham patch. Additionally, pyloric exclusion and gastrojejunostomy were performed. Following these interventions, the patient remained symptom-free without recurrent issues for a number of years. 

**Figure 1:**
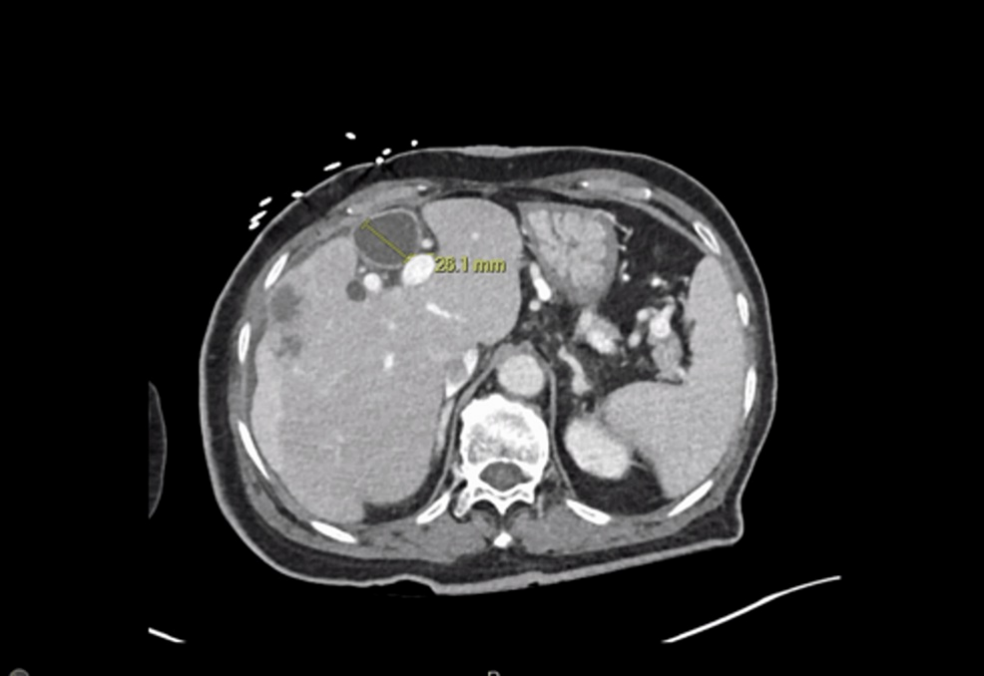
Axial CT of the abdomen with contrast shows dilation of the mid-distal CBD (26 mm). CT: computed tomography; CBD: common bile duct

**Figure 2:**
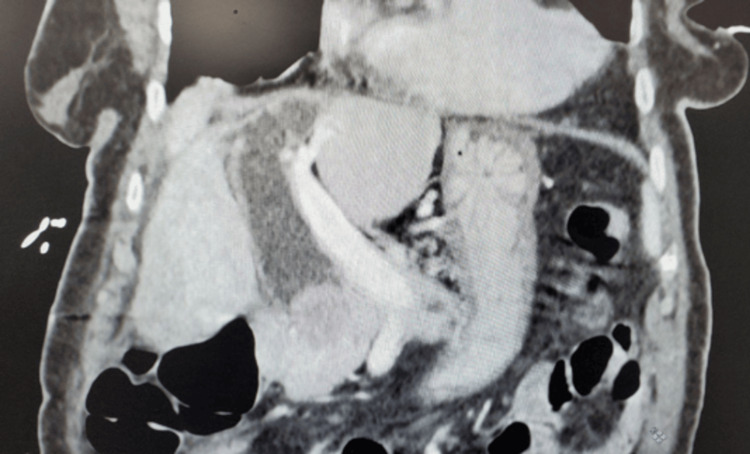
Coronal CT with contrast with large distal CBD stone measuring 24x27x33 mm. CT: computed tomography; CBD: common bile duct

However, the patient presented to the emergency department with a three-day history of right upper quadrant pain and fevers. Labs were notable for a total bilirubin of 2.2 mg/dL (reference: 0.2-1.4 mg/dL) and a serum alkaline phosphatase of 140 IU/L (reference: 36-112 IU/L). A computed tomography (CT) scan upon admission revealed a prominent 24x27x33 mm stone in the distal CBD, causing severe dilation (Figure 1 and Figure 2).

Additionally, an irregular cystic mass was observed in the right lobe of the liver. The patient exhibited signs of sepsis due to presumed cholangitis, presenting with an initial fever of 102.7°F, hypotension, and tachycardia. This complex clinical scenario demanded urgent evaluation and management to address the acute cholangitis and its associated complications.

Upon the patient's admission, the initial focus of management was directed toward restoring hemodynamic stability. This involved the administration of bolus intravenous normal saline and the initiation of empiric antibiotics tailored for acute cholangitis.

**Figure 3:**
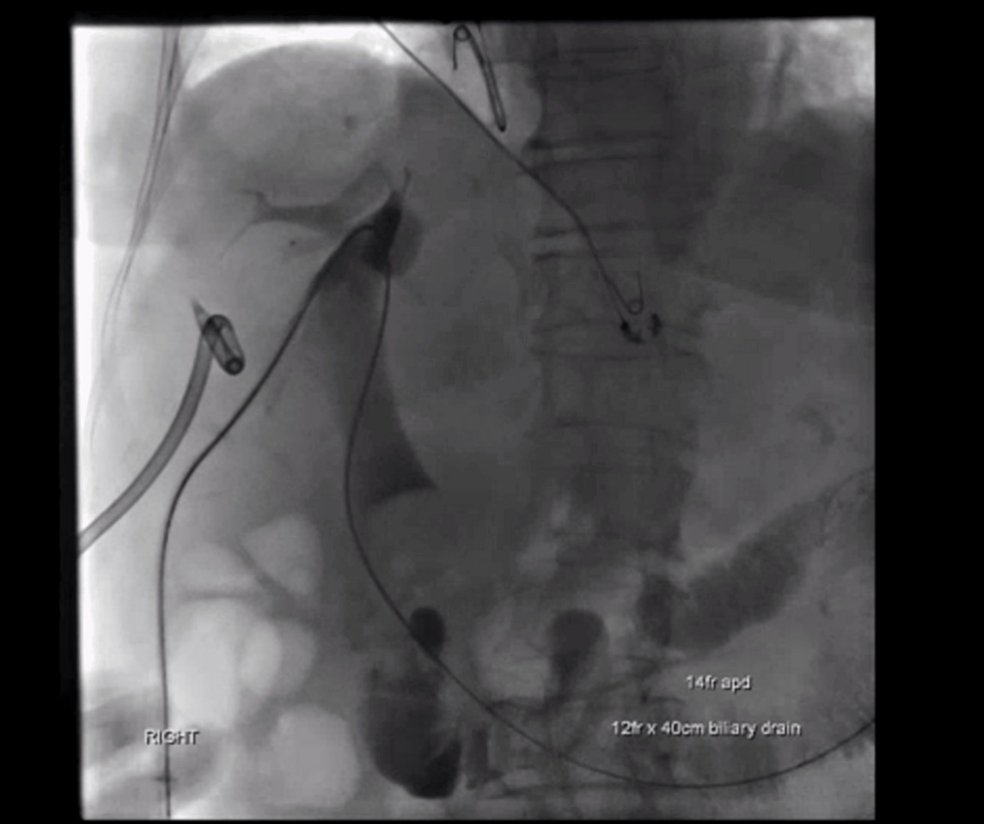
A 12F right internal-external biliary drain and 14F right liver lesion drain were placed under transhepatic cholangiography.

The chosen approach encompassed a series of interventions aimed at controlling the source of infection and decompressing the biliary tree. A percutaneous transhepatic biliary drainage (PTBD) procedure was performed, involving the insertion of a 12F internal-external biliary drain. The distal pigtail was carefully placed in the jejunum to optimize drainage. Simultaneously, a 14F liver lesion drain was strategically positioned (Figure 3). This resulted in a markedly reduced bilirubin level as of post-op day 1.

**Figure 4:**
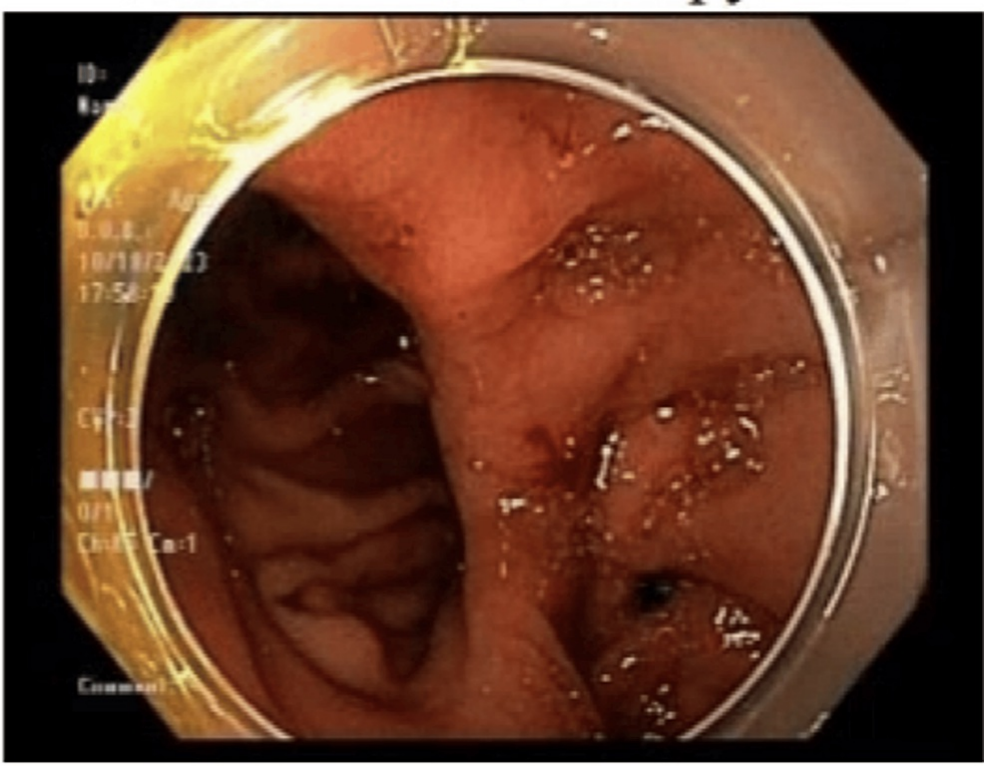
Visualization of gastrojejunostomy anastomosis.

Subsequently, an ERCP was undertaken. An endoscope was inserted, and gastrojejunostomy anastomosis was visualized to be intact (Figure 4). Balloon dilation of the CBD was executed, and access to the duodenal bulb was achieved through the pylorus; however, there was notable stenosis of the pylorus.

**Figure 5:**
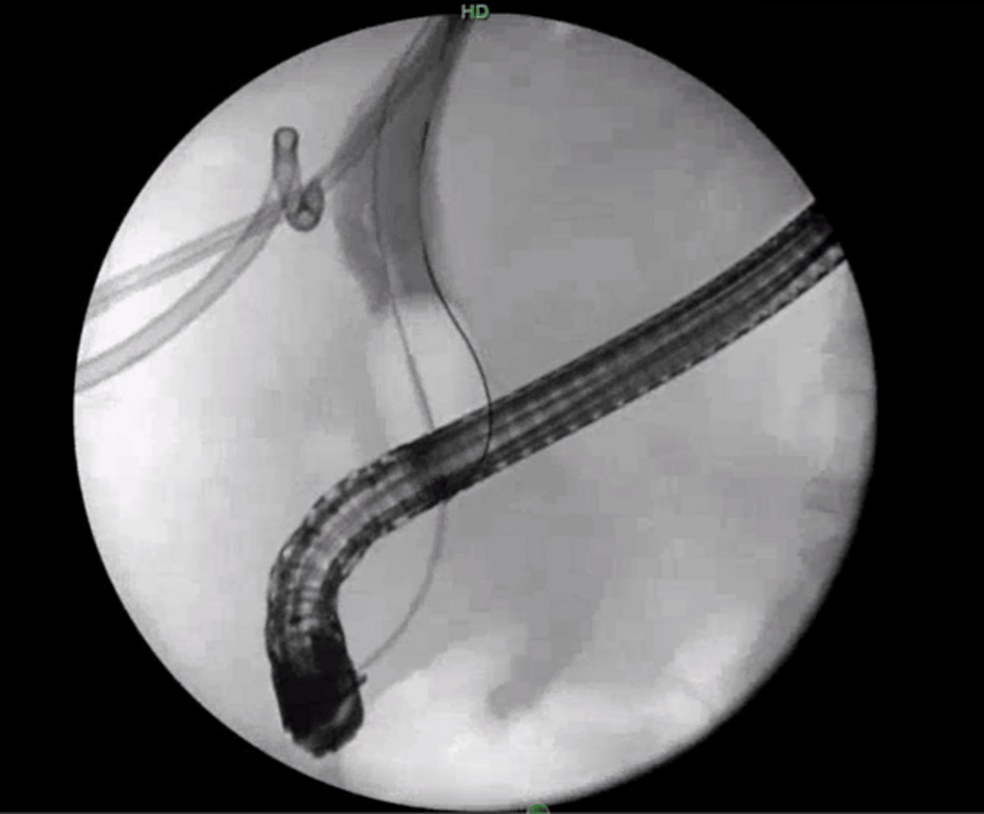
ERCP showing a blockage of the CBD. ERCP: endoscopic retrograde cholangiopancreatography; CBD: common bile duct

An occlusion cholangiogram revealed significant dilation of the CBD and common hepatic duct (CHD), measuring 20 mm proximally and 30 mm distally (Figure 5). Notably, the intrahepatic ducts appeared within normal limits.

**Figure 6:**
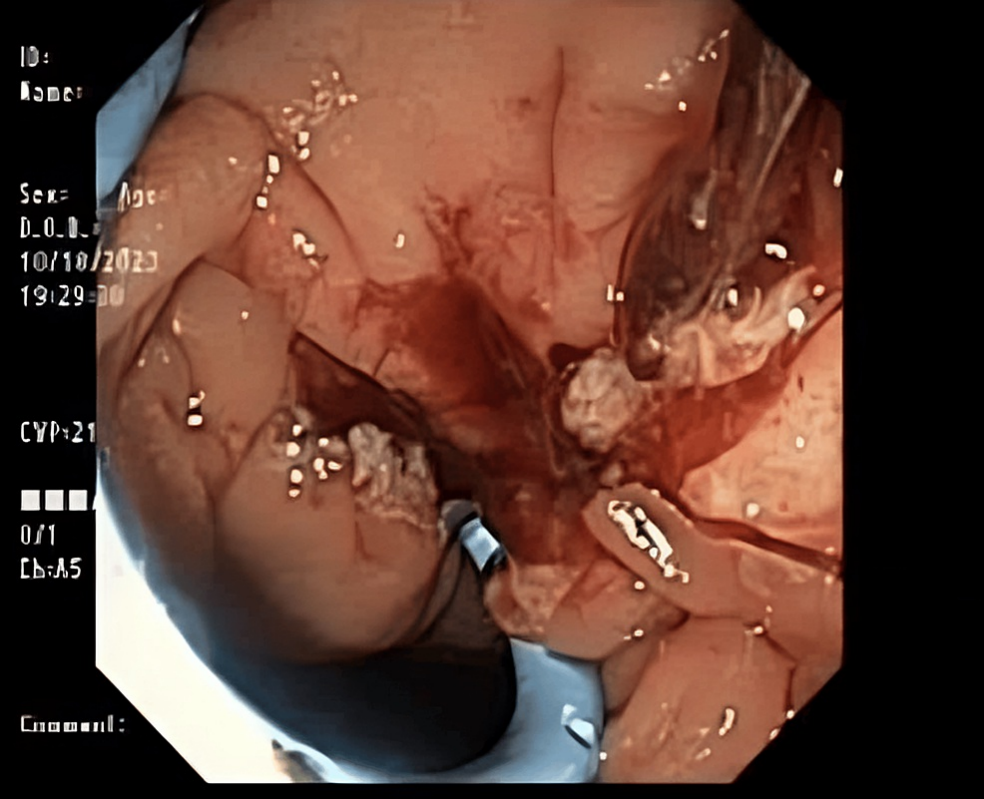
Internal biliary plastic stent placed in the CBD. CBD: common bile duct

To address the obstruction, a biliary sphincterotomy was performed. Visual inspection confirmed the presence of a 30 mm biliary stone within the CBD. An initial attempt to deploy a 9-12 mm biliary stone retrieval balloon proved unsuccessful. Consequently, a decision was made to perform a sphincteroplasty. Pressure was sustained for one minute in an effort to facilitate dilation. Despite these efforts, the attempt to remove the stone using the biliary stone retrieval balloon was still met with failure. To ensure adequate drainage of the biliary tree, an internal biliary stent was meticulously placed, serving as a conduit for continuous drainage and alleviating the obstructive symptoms experienced by the patient (Figure 6).

A second ERCP was planned due to a large stone in the CBD. The plastic CBD stent was removed to allow for better access to the stone. Cannulation of the biliary duct was accomplished with a sphincterotome revealing dilation of the CBD and CHD up to 30 mm. The stone, larger than 30 mm and located mid-CBD, could not be bypassed by the plastic double pigtail stents. 

Thus, lithotripsy was chosen as the treatment strategy. Using the SpyGlass cholangioscopy (Boston Scientific, Marlborough, MA) and an electrohydraulic lithotripsy (EHL) catheter, the stone was successfully fragmented under direct visualization.

After the lithotripsy, a stone retrieval balloon was used to clear the CBD. A subsequent cholangioscopy confirmed the removal of all calculi from the biliary tree.

Comparing alkaline phosphatase, alanine transaminases (ALTs), and aspartate transaminases (ASTs), we saw a significant downtrend between day 1 pre procedure and day 1 post lithotripsy and stone retrieval (Table 1). The downgoing trend of liver enzyme levels, total bilirubin, and clinical improvement including resolution of fever, jaundice, and improved general appearance assures us that the obstruction was cleared and is consistent with evidence of resolving cholangitis.

**Table 1:** Selected lab values pre procedure and post procedure of ERCP with lithotripsy. ERCP: endoscopic retrograde cholangiopancreatography

Lab value	Pre procedure	Post-op day 1 ERCP with lithotripsy	Standard reference ranges
Total bilirubin	2.2	1	0.2-1.4 mg/dL
Direct bilirubin	1.3	0.4	0-0.2 mg/dL
Alkaline phosphatase	141	70	36-112 IU/L
Alanine transaminase	100	71	6-47 IU/L
Aspartate transaminase	97	54	10-33 IU/L

## Discussion

Ascending cholangitis is a serious and potentially life-threatening condition characterized by inflammation and infection of the biliary tree. This condition typically arises from bacterial contamination of the biliary system due to obstruction, often caused by choledocholithiasis. The development of gallstones is influenced by multiple factors, with established risk elements encompassing being over the age of 40, being female, leading a sedentary lifestyle, consuming alcohol or tobacco, and conditions such as liver cirrhosis or Crohn's disease [1]. The obstruction of the CBD leads to stasis of bile, providing a favorable environment for bacterial overgrowth, particularly of enteric organisms such as *Escherichia coli*, *Klebsiella*, and *Enterococcus* species [2]. Laboratory findings commonly reveal elevated liver enzymes, including alkaline phosphatase, along with elevated levels of conjugated bilirubin. Leukocytosis is often present, indicating an inflammatory response. Prompt management is crucial to prevent septic complications and systemic deterioration. Early administration of broad-spectrum antibiotics is essential to cover both gram-negative and anaerobic bacteria. Furthermore, urgent biliary decompression through ERCP or percutaneous transhepatic cholangiography (PTC) is necessary to relieve the biliary obstruction and facilitate drainage [3].

Firstly, the presentation underscores the necessity of a meticulous review of the patient's surgical history before undertaking interventional procedures. The past cholecystectomy, hepatectomy, duodenal repair with a Graham patch, and altered gastrointestinal continuity due to gastrojejunostomy significantly increased the complexity of the endoscopic interventions. An awareness of such anatomical changes is paramount to avoid perioperative complications and to plan the most appropriate approach. The occurrence of iatrogenic duodenal perforation during the initial ERCP attempts is a sobering reminder of the risks associated with endoscopic procedures, particularly in patients with previous abdominal surgeries [4]. This case emphasizes the importance of vigilance and the need for surgical backup during high-risk procedures.

The patient's complex surgical history raised concerns regarding the optimal approach to address the acute cholangitis and associated complications. The initial management focused on stabilizing the patient's hemodynamic status and initiating empiric antibiotic therapy tailored for acute cholangitis [5]. A multidisciplinary team collaborated to devise a comprehensive treatment strategy, which involved a series of interventions to control the source of infection and relieve biliary obstruction. PTBD was performed, along with the placement of an internal-external biliary drain and a liver lesion drain to optimize drainage and decrease bilirubin levels. ERCP was subsequently employed to address the biliary obstruction.

During the first ERCP attempt, it was clear that a creative endoscopic approach was needed due to the anatomical considerations imposed for this case. Accessing the biliary limb became challenging with the previous surgical history of a gastrojejunostomy. While the stenosed pylorus was navigable, accessing the biliary limb was still challenging. An alternative approach, like enteroscopy-assisted ERCP (similar to that used in Roux-en-Y gastric bypass patients), was considered but not pursued due to its limitations in endoscope maneuverability and difficulty in identifying and cannulating the papilla [6]. Eventually, a follow-up ERCP with lithotripsy via SpyGlass cholangioscopy and an EHL catheter successfully fragmented and cleared a large CBD stone [7]. This outcome reflects the team's meticulous approach and careful consideration of the patient's advanced age and frail condition, highlighting the importance of balancing aggressive management with the risks of further interventions in elderly patients with complex surgical histories.

## Conclusions

This case underscores the critical challenges associated with managing ascending cholangitis in an elderly patient with a complex surgical history and altered anatomy. The patient's acute presentation with a large CBD stone necessitated a multidisciplinary approach, incorporating advanced endoscopic techniques and meticulous decision-making to ensure optimal outcomes. Timely administration of broad-spectrum antibiotics and urgent biliary decompression through PTBD and ERCP also played a pivotal role in addressing the biliary obstruction and resolving the septic complications. Successful implementation of sphincterotomy, sphincteroplasty, and lithotripsy using SpyGlass cholangioscopy and EHL resulted in the fragmentation and clearance of the CBD stone, as indicated by the significant downtrend in liver function test (LFTs) and bilirubin levels, along with the resolution of the patient's clinical symptoms.

Careful consideration of the patient's age and frailty was paramount in balancing the aggressiveness of the interventions with the potential risks associated with further procedures. The importance of individualized patient care plays such a crucial role for a multidisciplinary team in navigating the complexities of managing intricate cases, ensuring optimal patient outcomes and quality of life.
